# The role of nutritional risk evaluation in predicting adverse outcomes among patients with severe COVID-19 in Vietnam

**DOI:** 10.3389/fnut.2023.1245816

**Published:** 2023-10-05

**Authors:** Lan Huong Thi Nguyen, Anh Kim Dang, Tien Viet Tran, Hai Thanh Phan, Dao Anh Thi Doan, Lien Bao Thi Nguyen, Anh Minh Tran, Tung Dinh Do, Thang Ba Nguyen, Tien Thanh Nguyen, Binh Huy Nguyen, Huong Thi Le

**Affiliations:** ^1^School of Preventive Medicine and Public Health, Hanoi Medical University, Hanoi, Vietnam; ^2^Department of Nutrition, Saint Paul General Hospital, Hanoi, Vietnam; ^3^Queensland Alliance for Environmental Health Sciences (QAEHS), The University of Queensland, Woolloongabba, QLD, Australia; ^4^Department of Nutrition, Thanh Nhan General Hospital, Hanoi, Vietnam; ^5^Intensive Care Unit and Poison Control, Saint Paul General Hospital, Hanoi, Vietnam; ^6^Saint Paul General Hospital, Hanoi, Vietnam; ^7^Department of Physiology, Hanoi Medical University, Hanoi, Vietnam

**Keywords:** nutritional status, COVID-19, Vietnam, Nutritional Risk Screening (NRS), The Global Leadership Initiative on Malnutrition (GLIM), Prognostic Nutritional Index (PNI)

## Abstract

**Introduction:**

As sufficient nutrition helps alleviate catabolic stress and modulate the systemic inflammatory response of the body, it plays an indispensable role in the good prognosis of critically ill patients. Thus, this study aimed to investigate the malnutrition of patients with severe COVID-19 and its association with adverse treatment outcomes.

**Methods:**

We conducted a retrospective cross-sectional study in two provincial hospitals in Hanoi from February to April 2022. Participants were patients with severe COVID-19 admitted to the Intensive Care Unit (ICU). Malnutrition risk were evaluated by Nutritional Risk Screening-2002 (NRS), Global Leadership Initiative on Malnutrition (GLIM), Prognostic Nutritional Index (PNI), and the adverse prognosis was assessed by Acute Physiology and Chronic Health Evaluation II (APACHE II). The multivariate receiver-operating characteristic (ROC) curve was applied to estimate the predictive ability of those criteria regarding worse treatment results.

**Results:**

The percentages of malnutrition measured by NRS, GLIM, PNI, and BMI were 62.6, 51.5, 42.9, and 16.6%, respectively. Patients with more severe malnutrition assessed by GLIM, PNI, and having above target fasting blood glucose (FBG) (≥10.0 mmol/L) were more likely to have higher APACHE scores. PNI had a better diagnostic performance than NRS and BMI (AUC = 0.84, 0.81, and 0.82, respectively). In addition, FBG revealed a good prognostic implication (AUC = 0.84).

**Conclusion:**

A relatively high percentage of patients experienced moderate and severe malnutrition regardless of screening tools. Individuals at higher risk of malnutrition and high FBG were predicted to have more adverse treatment outcomes. It is recommended that nutritional screening should be conducted regularly, and personalizing nutritional care strategies is necessary to meet patients’ nutrient demands and prevent other nutrition-related complications.

## 1. Introduction

It is well-documented that adequate nutrition is indispensable for a good prognosis of critically ill patients (1–3). Malnutrition can exacerbate the body’s metabolic stress and systemic inflammatory response, triggering organ failure, other infections, and prolonged hospital stays (1). Severe conditions accompanied by a hyper-metabolic state and immune responses can contribute to cytokine storms, speeding up the process of proteolysis and undernutrition (2). Thus, appropriate medical nutrition therapy is necessary to alleviate catabolic stress, modulate immune responses, and prevent the deterioration of the body’s lean mass, significantly improving treatment outcomes (1, 3). These nutritional therapies cover the evaluation of dietary needs, patients’ preference for calorie and protein intake, and route of administration to confine malnutrition (4). COVID-19 is an acute inflammatory condition and can lead to refractory respiratory failure, shock with multi-organ failure, and cardiac and neurologic causes (5). Sufficient nutritional care can also assist people suffering from severe COVID-19 withstanding critical conditions.

Previous studies showed that undernutrition has been recognized as an underlying mortality risk factor among COVID-19 patients (6, 7). Thus, nutrition risk screening is recommended as a compulsory examination for COVID-19 patients at hospital admission. The European Society for Clinical Nutrition and Metabolism (ESPEN) provides expert consensus guidance on managing nutritional risk among COVID-19 patients to mitigate malnutrition’s impact on morbidity and mortality (8). Malnutrition Universal Screening Tool (MUST), Nutritional Risk Screening-2002 (NRS-2002), and Global Leadership Initiative on Malnutrition (GLIM) are suggested by the ESPEN to identify the risk and presence of malnutrition among COVID-19 patients (8). However, the accuracy of anthropometric indices to evaluate the nutritional status of patients can be uncertain due to overhydration or lack of appropriate measurements. The precise body weight and height of patients in the ICU can be difficult to measure because of body composition changes, fluid administration, or increased vasopermeability induced by serious illness (9). In addition, the poor condition of patients with critical illness may progress rapidly and heterogeneous. Therefore, a quick and easy-to-obtain nutritional assessment tool is preferred to determine the severity and prognosis of the disease, compared to previous instruments (10). Albumin is an important indicator reflecting protein deficiency and malnutrition, while lymphocytes are strongly relevant to patients’ immune status (11). Prognostic Nutritional Index (PNI), a scale combining albumin concentration and total lymphocyte count, is recommended in previous studies to assess the immune-nutritional situation of patients (6, 12).

In the context of Vietnam, by April 2022, the country experienced the fourth wave of COVID-19 infection and had more than 10.5 million cases, with thousands of severe cases (13). All national COVID-19 treatment guidelines agree that nutritional screening is mandatory in all healthcare facilities with COVID-19 patients upon admission. In a previous initial study, more than half of COVID-19 patients admitted to the Intensive Care Unit (ICU) reported undernutrition using GLIM criteria (65%) (14). In further studies, assessing the nutritional status of severely ill COVID-19 patients using different scales is essential for a multi-dimensional perspective. Furthermore, the association between nutritional risk and adverse outcomes requires a more thorough investigation to propose timely interventions. Therefore, the primary aim of the study was to determine the malnutrition of severe COVID-19 patients using different screening tools and its relation to adverse treatment results. Secondly, we also evaluate the applied criteria’s diagnostic ability to predict the study subjects’ negative conditions.

## 2. Methods

### 2.1. Study setting and participants

We conducted a cross-sectional study on patients hospitalized for severe COVID-19 in two provincial hospitals in Hanoi from February to April 2022. This study design was appropriate because the percentage of malnutrition in severe COVID-19 patients was assessed quickly, and the relationship between poor-nourished and severe illness conditions was determined so that nutritional interventions could be given precisely. Patients were recruited according to the following criteria: (1) aged 18 years old or older; (2) being positive with SARS-CoV-2 confirmed by Reverse Transcriptase Polymerase Chain Reaction (RT-PCR); (3) diagnosed with moderate or severe COVID-19 by the classification of Vietnamese Ministry of Health; (4) having nutrition status assessment at admission to ICU. We excluded participants if the nutritional evaluation was not sufficiently documented or if they were pregnant or breastfeeding women.

### 2.2. Sample size and sampling technique

We used the sample size formula for estimating a proportion. Data from a previous study showed that 67.3% of patients admitted to the ICU had malnutrition using the Nutritional Risk Screening scale (7). Z of ( $1-\frac{\alpha }{2})$ for a 95% confidence interval was 1.96, ε = 0.1:

$$n=Z{{\;}_{\left(1-\frac{\alpha }{2}\right)}}^{2}\;x\frac{\left(1-p\right)}{p\varepsilon^{2}}$$

Thus, the calculated sample size was 154 patients. Finally, we collected data from 163 patients hospitalized during the research time in specialized COVID-19 treatment departments in the mentioned study settings. Because the number of patients with serious COVID-19 conditions admitted to the ICU was less than those with light symptoms in other departments, we applied convenience sampling to ensure the study sample size. Patients admitted to the ICU meeting the selection criteria were invited to participate in the study.

### 2.3. Data collection

#### 2.3.1. Nutritional risk assessment

The weight and height of patients were systematically measured at admission to the ICU. We recorded the information regarding patients’ weight loss via patients self-reported or their main caregivers if they could not provide the answers. To screen for malnutrition, we applied several international standard scales, including Nutritional Risk Screening-2002 (NRS), Global Leadership Initiative on Malnutrition (GLIM), Body Mass Index (BMI), and Prognostic Nutritional Index (PNI). BMI and NRS are mandatory nutritional screening tools conducted at hospital admission and performed by health personnel. GLIM is the scale suggested by the ESPEN to determine malnutrition among COVID-19 patients. In addition, PNI was used to reflect the immune-nutritional status of patients along with other screening tools.

NRS has been validated and widely used in previous studies, and it was proven reliable if managed by trained health personnel (15). This tool incorporates four questions to pre-screen the malnutrition risk for patients (16), including (1) a BMI <20.5 kg/m^2^; (2) weight loss during the last 3 months; (3) reduction of food intake in the past week; and (4) disease severity. A final screening would be evaluated if any parameter was marked as positive. In each category (impaired nutritional status and disease severity), a score from 0 to 3 is given. Age ≥70 years is also considered a risk factor, which adds 1 point to the score. Finally, a total score ≥ 3 points explains that patients are undernutrition or at risk of undernutrition, and nutritional care plans are required (16).

GLIM is a global consensus that focuses on diagnosing malnutrition in adults and categorizing the severity level (17). Phenotypic criteria include weight loss, low BMI, and reduced muscle mass, while the etiologic measure is determined by reduced food intake, assimilation, and inflammation. A patient with at least one phenotypic and etiologic parameter is diagnosed with undernutrition (17). The severity level of phenotypic items is used to categorize malnutrition as moderate (grade 1) or severe (grade 2) (17).

PNI has been widely applied in hospitals to reflect patients’ immunological and nutritional status (18). This tool is calculated by the following equation: (10 × serum albumin (g/dL)) + (0.005 × total lymphocyte count (mm^3^)) (19). The threshold of > 38, 35–38 and < 35 was used to define the normal, moderate and severe risk of undernutrition, respectively (19). PNI was first suggested to assess the nutritional status of patients with general surgery (18) and is considered a helpful parameter to predict treatment outcomes of those with certain kinds of cancer (20). Previous studies revealed that PNI could be used to determine the immune-nutritional status of COVID-19 patients and predict the severity of COVID-19 (19, 20).

#### 2.3.2. Clinical characteristics and laboratory measurements

We collected information about the clinical features of patients, which covered the severity of COVID-19, length of ICU stay, several chronic comorbidities (diabetes mellitus, chronic respiratory disease, cardiovascular disease, and others), types of used medication (antiviral medicines, corticosteroids, and antibiotics). Regarding the severity of the disease, we divided it into three levels, including moderate (using high-flow nasal oxygen), severe (non-invasive ventilation through a face mask to eliminate the need for the endotracheal airway) and critically ill (Invasive mechanical ventilation with the use of vasopressors). The classification was based on the professional opinions of the specialists and the treatment guidelines for COVID-19 patients released by the Ministry of Health (21). All patients underwent physical examinations at admission to measure systolic (SBP) and diastolic blood pressure (DBP), body temperature, and respiration rate. Main arterial pressure (MAP) was defined as (SBP + 2 × DBP)/3. These clinical characteristics were important to evaluate the severity of the COVID-19 progression from experts’ perspectives and scoring the Acute Physiology and Chronic Health Evaluation II.

Laboratory measurements were carried out in a standard manner by collecting fasting venous blood samples during hospitalization. The evaluated parameters included Hemoglobin (g/L), Hematocrit (%), Total lymphocyte count (×109/L), Total protein (g/L), Albumin (g/L), CRP (mg/dL), Fasting blood glucose (FBG) (mmol/L), Creatinine (μmol/L), Aspartate transaminase (U/L), Alanine transaminase (U/L), Sodium (mEq/L), Potassium (mEq/L), Chloride (mEq/L). We classified FBG into two groups according to the American Diabetes Association (ADA) “Standards of Medical Care in Diabetes,” in which the above target was defined if FBG ≥ 10.0 mmol/L and on target/ below target was 7.8—under 10.0 mmol/L or < 7.8 mmol/L, respectively (22).

In addition, to predict the adverse outcomes of patients in the ICU, the clinical characteristics in combination with sub-clinical characteristics were vital. Therefore, the APACHE II score, a well-used classification system for grading disease severity using these features, was applied (23). APACHE II consists of three categories (12 acute physiology characteristics, previous chronic health and age) that yield the final score ranging from 0 to 71 (24). This is an important prognostic marker for COVID-19 patients, with a higher score corresponding to a higher risk of adverse outcomes and mortality. A cut-off point of 13 was used to define those with a higher risk of adverse outcomes and mortality with a good discriminative ability (25).

#### 2.3.3. General characteristics and health risk behaviors

Several health risk behaviors can increase the critical level of COVID-19 patients; for instance, smoking may damage the lungs and using alcohol can affect body organs. Besides, the COVID-19 vaccine is shown to mitigate the severity and long duration of COVID-19 symptoms. Therefore, in this study, data on demographic characteristics and health risk behaviors, including gender, age, vaccination status, alcohol use, and smoking, were retrieved from medical records.

### 2.4. Data analysis

All statistical analyses were conducted using STATA 15 (StataCorp, College Station, TX, United States). We classified the sample into three groups based on COVID-19 severity level (moderate, severe and critically ill). Continuous variables were described as mean ± standard deviation (SD) and the Kruskal-Wallis test was applied to compare the differences between the three groups based on the severity level. Categorical variables were expressed as frequency and percentage (n, %) and compared using Fisher’s exact test (if >20% of cells had expected frequencies <5) or Chi-squared test. We considered the statistical significance at value of *p* < 0.05. The multivariate receiver-operating characteristic (ROC) curve and the area under the curve (AUC) were used to evaluate the performance of nutritional assessment scales to diagnose detrimental outcomes of patients with COVID-19. Multivariate logistic regression models were carried out to estimate the association between different nutritional evaluation tools and APACHE score by the coefficient (Coef) and 95% confidence intervals (95%CI). To select variables of the multivariate logistic regression models, we started with the variables of known clinical importance. Any variables with a value of *p* < 0.25 from the univariate test were appropriate candidates for the multivariate analysis. We also used “VIF” command in the software to test for multicollinearity.

### 2.5. Research ethics

The study was conducted with the approval of the committee of Saint Paul Hospital, code number 645/QĐ-BVĐKXP. We prepared the written informed consent to ask permission from patients and their main caregivers to involve them in the study. All data retrieved from medical records were used for research purposes only. Patients can reject or withdraw from the research at any time. All patients’ names were not collected, and each was coded with an identifier. All information was kept confidential and encrypted according to the research questionnaire. Only the research team leader had access to research data.

## 3. Results

One-hundred sixty-three patients were involved in the final analysis. There were 27 patients with moderate conditions, 100 with severe COVID-19, and 36 patients experiencing critical illness. Table 1 presents the general characteristics of patients defined by different severity levels of COVID-19. Nearly half of the participants were men (45.4%), and the mean age was 68 (SD = 18). The number of patients not having the COVID-19 vaccine in severe and critically ill groups is significantly higher than in the moderate group (56.0 and 55.6% compared to 33.33%, respectively). In addition, the percentage of patients with diabetes mellitus in the moderate group is significantly lower than that of the critically ill patients (37.0% versus 55.6%, respectively). 51.5% of the participants reported having a history of cardiovascular diseases. The mean duration of treatment was 10.8 days (SD = 5).

**Table 1:** Characteristics of the patients according to severity levels of COVID-19.

	Moderate (*n* = 27)		Severe (*n* = 100)		Critically ill (*n* = 36)		Total (*n* = 163)		*p*-value
	*n*	%	*n*	%	*n*	%	*n*	%	
Gender									
Men	14	51.9	43	43.0	17	47.2	74	45.4	0.69
Women	13	48.1	57	57.0	19	52.8	89	54.6	
Number of COVID-19 vaccine doses									
Zero	9	33.3	56	56.0	20	55.6	85	52.2	<0.01
One	0	0.0	8	8.0	8	22.2	16	9.8	
Two and above	18	66.7	49	49.0	19	52.8	86	52.8	
Diabetes mellitus	10	37.0	36	36.0	20	55.6	66	40.5	0.04
Chronic respiratory disease	0	0.0	10	10.0	2	5.6	12	7.4	0.22
Cardiovascular disease	15	55.6	54	54.0	15	41.7	84	51.5	0.40
Other chronic diseases	4	14.8	16	16.0	8	22.2	28	17.2	0.66
Alcohol use									
Non-using or former drinkers	24	88.9	98	98.0	34	94.4	156	95.7	0.06
Currently using	3	11.1	2	2.0	2	5.6	7	4.3	
Smoking									
Non-smoking or former smokers	26	96.3	96	96.0	30	83.3	152	93.3	0.04
Currently smoking	1	3.7	4	4.0	6	16.7	11	6.7	
	Mean	SD	Mean	SD	Mean	SD	Mean	SD	
Age (years)	71	14.0	67	19.0	69	18.0	68	18.0	0.76
Length of ICU stay (days)	10.7	5.1	11.0	5.3	10.2	4.2	10.8	5.0	0.88

The information regarding laboratory characteristics of COVID-19 patients is shown in Table 2. More patients with severe and critical conditions were treated with corticosteroids and antibiotics. The average body weight of participants was 54.9 kg (SD = 9.0). The hemoglobin and total lymphocyte count level in the moderate group was significantly lower than in the other groups (*p* = 0.04 vs. 0.01, respectively). Regarding nutritional parameters in Table 2, albumin was lower with the increasing severity of COVID-19. However, the result was not statistically significant. To measure the level of adverse outcomes, the APACHE scores in moderate, severe, and critically ill groups were 5.8, 7.1, and 8.2, respectively.

**Table 2:** Laboratory measurements of the patients according to severity levels of COVID-19.

	Moderate (*n* = 27)		Severe (*n* = 100)		Critically ill (*n* = 36)		Total (*n* = 163)		*p*-value
	*n*	%	*n*	%	*n*	%	*n*	%	
Medication used in treatment									
Antiviral medicines	18	66.7	54	54.0	16	44.4	88	54.0	0.22
Corticosteroids	16	59.3	78	78.0	30	83.3	124	76.1	0.04
Antibiotics	17	63.0	86	86.0	36	100.0	139	85.3	<0.01
	Mean	SD	Mean	SD	Mean	SD	Mean	SD	
Weight (kg)	56.9	9.1	54.3	8.8	55	9.4	54.9	9.0	0.39
Mean arterial pressure (MAP)	92.7	8.7	90.7	9.5	88.6	9.2	90.6	9.3	0.16
Body temperature (°C)	37.3	0.7	37.2	0.8	36.9	0.5	37.2	0.8	0.03
Respiration rate	24.1	4.8	23.6	3.9	25.7	4.6	24.1	4.3	0.15
Hemoglobin (g/L)	128.9	10.4	124.4	21.2	114.3	38.5	123.0	25.0	0.04
Hematocrit (%)	38.1	3.7	37.2	6.7	35.2	9.0	36.9	7.0	0.60
Total lymphocyte count, ×10^9^/L	2.2	2.0	1.5	1.3	1.0	0.5	1.5	1.0	0.01
Total protein (g/L)	60.8	7.5	61.5	6.4	63.4	6.1	61.8	6.5	0.02
Albumin (g/L)	34.9	4.2	33.5	4.6	33.4	3.2	33.7	4.3	0.14
CRP (mg/dL)	10.9	7.5	11.2	7.0	11.6	5.0	11.2	6.7	0.46
Fasting blood glucose (mmol/L)	9.4	3.1	11.0	4.7	10.4	4.6	10.6	4.5	0.44
Creatinine (μmol/L)	84.9	16.0	84.5	27.1	108.7	67.2	89.9	39.6	0.13
Aspartate transaminase (U/L)	47.3	35.8	60.0	57.0	107.2	89.0	68.3	30.1	<0.01
Alanine transaminase (U/L)	34.3	24.0	40.2	30.8	50.0	41.8	41.4	32.8	0.28
Sodium (mEq/L)	134.9	2.5	133.2	7.3	133.1	7.9	133.4	6.9	0.04
Potassium (mEq/L)	3.7	0.3	3.9	0.7	4.1	0.6	3.9	0.6	<0.01
Chloride (mEq/L)	100	3.7	96.2	19.8	100.3	8.2	97.7	16.1	0.45
APACHE score	5.8	2.3	7.1	3.5	8.2	3.2	7.2	3.3	<0.01

APACHE, Acute Physiology and Chronic Health Evaluation scale—a classification system for grading disease severity.

Table 3 illustrates the nutritional status of patients with COVID-19. Overall, the percentages of malnutrition measured by NRS, GLIM, PNI, and BMI were 62.6, 51.5, 42.9, and 16.6%, respectively. The NRS score in the severe and critical groups was significantly higher than in the moderate group (3.1, 3.0, and 2.4, respectively). By contrast, the PNI scores in moderate, severe and critically ill groups were 46.2, 41.0, and 38.1, respectively. The mean NRS, PNI and BMI values were 3.0 (SD = 1.3), 41.3 (SD = 8.9), and 22.1 (SD = 3.7), respectively.

**Table 3:** Nutritional status of the patients according to different severity level of COVID-19.

	Moderate (*n* = 27)		Severe (*n* = 100)		Critically ill (*n* = 36)		Total (*n* = 163)		*p*-value
	*n*	%	*n*	%	*n*	%	*n*	%	
NRS score (Mean, SD)	2.4	1.0	3.1	1.2	3.0	1.4	3.0	1.3	0.03
Malnutrition risk assessed by NRS									
Normal	15	55.6	34	34.0	12	33.3	61	37.4	0.10
Under-nutrition	12	44.4	66	66.0	24	66.7	102	62.6	
Malnutrition risk assessed by GLIM									
Normal	16	59.3	49	49.0	14	38.9	79	48.5	0.58
Moderate	10	37.0	42	42.0	19	52.8	71	43.5	
Severe	1	3.7	9	9.0	3	8.3	13	8.0	
PNI score (Mean, SD)	46.2	13.0	41.0	8.4	38.1	4.0	41.3	8.9	<0.01
Malnutrition risk assessed by PNI									
Normal	19	70.4	56	56.0	18	50.0	93	57.1	0.47
Moderate	6	22.2	28	28.0	10	27.8	44	27.0	
Severe	2	7.4	16	16.0	8	22.2	26	15.9	
BMI score (Mean, SD)	22.8	2.9	22	3.9	22	3.7	22.1	3.7	0.56
Malnutrition risk assessed by BMI									
Normal	26	96.3	80	80.0	30	83.3	136	83.4	0.13
Under-nutrition	1	3.7	20	20.0	6	16.7	27	16.6	

NRS, Nutrition Risk Screening scale; GLIM, Global Leadership Initiative on Malnutrition; PNI, Prognostic Nutritional Index; BMI, Body Mass Index.

**Figure 1:**
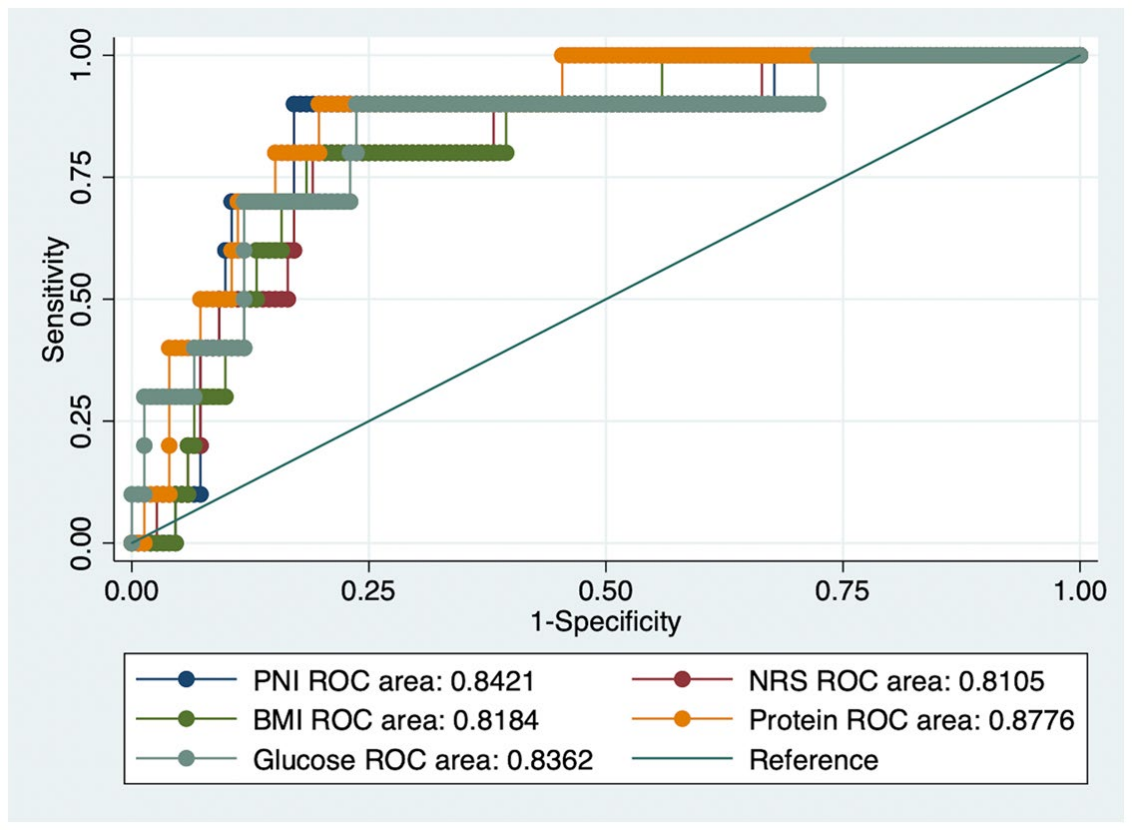
The receiver operating characteristic (ROC) curves for (1) Prognostic Nutritional Index (PNI); (2) Nutrition Risk Screening (NRS); (3) Body Mass Index (BMI); (4) Total Blood Protein; (5) Fasting blood glucose values to detect severe outcomes using the Acute Physiology and Chronic Health Evaluation scale (APACHE).

Applying the APACHE cut-off of 13, people having adverse outcomes accounted for 6.1% of all patients. Figure 1 depicts the areas of (ROC) curves for different nutritional assessment scales to predict the negative outcomes of COVID-19. A ROC curve is a figure presenting the performance of a classification characteristic at all classification thresholds. It shows that PNI, NRS, and BMI had a good diagnostic performance (ROC area = 0.84, 0.81, and 0.82, respectively). In addition, total blood protein and FBG also revealed a good prognostic implication (ROC area = 0.88 and 0.84, respectively).

Table 4 presents the adjusted multivariate logistic regression models determining the association between malnutrition and the risk of experiencing adverse health outcomes among patients with severe COVID-19 conditions. There were four models, including NRS, GLIM, PNI, and FBG, adjusted by age, gender, duration of treatment, the severity of COVID-19, diabetes mellitus, chronic respiratory disease, cardiovascular disease, alcohol use, smoking, and number of COVID-19 vaccine doses. Regarding the GLIM scale, severely underweight people were more likely to have higher APACHE scores (Coef = 0.62; 95%CI = 0.43–1.97). Similarly, severe malnutrition assessed by the PNI tool was positively associated with a higher risk of having in-hospital mortality (Coef = 1.72; 95%CI = 0.71–2.74). Regarding co-morbidities, diabetes was strongly related to severe disease prognostic (Coef = 2.55; 95%CI = 0.96–4.13).

**Table 4:** Multivariate regression analysis to determine the role of nutritional risk score in predicting adverse outcomes among patients with severe COVID-19.

	APACHE score		
	Coef	95%CI	*p*-value
Model 1 Malnutrition risk assessed by NRS			
Normal	Ref		
Under-nutrition	0.23	−1.05; 0.59	0.58
Model 2 Malnutrition risk assessed by GLIM			
Normal	Ref		
Moderate	0.27	−0.48; 1.02	0.49
Severe	**0.62**	**0.43; 1.97**	**0.04**
Model 3 Malnutrition risk assessed by PNI			
Normal	Ref		
Moderate	0.03	−0.79; 0.84	0.80
Severe	**1.72**	**0.71; 2.74**	**<0.01**
Model 4 Fasting blood glucose group			
On target/ below target (<10.0 mmol/L)	Ref		
Above target (≥10.0 mmol/L)	**1.12**	**0.36; 1.89**	**<0.01**

Each model was adjusted by age, gender, duration of treatment, severity of COVID-19, Diabetes mellitus, Chronic respiratory disease, Cardiovascular disease, Alcohol use, Smoking, Number of COVID-19 vaccine doses. NRS, Nutrition Risk Screening scale; GLIM, Global Leadership Initiative on Malnutrition; PNI, Prognostic Nutritional Index; BMI, Body Mass Index; APACHE, Acute Physiology and Chronic Health Evaluation scale—a classification system for grading disease severity.Bold value means statistically significant (*p* < 0.05).

## 4. Discussion

To the best of our knowledge, this study is one of the first studies in Vietnam assessing the risk of malnutrition and predicting adverse outcomes among patients with severe COVID-19 in Vietnam. A relatively high percentage of patients experienced moderate and severe undernutrition, defined by NRS, GLIM, and PNI scales. PNI and FBG had a good diagnostic performance in predicting adverse outcomes using the APACHE II score. Findings also suggested that undernutrition patients assessed by GLIM and PNI, patients with above target FBG were more likely to have worse prognostic implications. Thus, these results shed light on evaluating and stratifying malnutrition risk in the better prognosis of severe COVID-19 patients in the ICU.

### 4.1. Nutritional assessment by NRS and GLIM scales

Using NRS and GLIM scales, a relatively high percentage of patients with severe COVID-19 underwent moderate to severe undernutrition. Nutritional risk screening is the first step for all hospitalized COVID-19 patients, and the NRS is a scale recommended by the Ministry of Health in Vietnam (26). NRS is also considered the first screening tool for malnutrition among patients with COVID-19 in several international guidelines (8), and a score ≥ 3 is related to a higher mortality risk (27). The percentage of undernutrition determined by the NRS tool ranged from 61.5 to 67.3% among COVID-19 patients admitted to ICU (7, 28).

The practical guidance of the European Society for Clinical Nutrition and Metabolism (ESPEN) also recommends GLIM, which consists of two approaching steps (defining at-risk patients and classifying the severity) for the diagnosis of malnutrition of patients with COVID-19 (8). A previous study validating the GLIM for diagnosing malnutrition in critically ill COVID-19 patients in 2021 revealed that 55% had malnutrition, while the figure for the SGA scale was 62.4% (29). The proportion of malnutrition assessed by BMI in our study was considerably lower than in other measurements. Previous studies also used BMI to define the nutritional status of COVID-19 patients that focus on obesity (7, 30). The disadvantage of BMI is not reflecting the weight or muscle mass loss within a reference time, as well as reduced dietary intake and critical status.

### 4.2. Nutritional assessment by PNI scale

Our study applied PNI to evaluate the immune-nutritional status of COVID-19 patients. The mean PNI score in this study is lower than previous studies conducted among Chinese COVID-19 patients [Mean = 48.5 (43.70–52.70) and Mean = 49.1; SD = 6.8] (19, 20). In addition, the score of PNI decreased with the condition of patients, from moderate to critically ill, aligning with a prior study (20). Furthermore, PNI has a high discriminatory power in detecting COVID-19 patients with adverse outcomes. In the literature, PNI was reported to have a good predictive ability in estimating mortality and disease severity among hospitalized COVID-19 patients (12, 20). Thus, when the information on weight loss and nutritional intake is insufficient, PNI can be applied as a simple index to objectively and effectively reflect the nutritional status of COVID-19 patients based on lymphocyte and albumin values.

Prior to COVID-19, PNI was emphasized to be associated with nutritional status and the response of the immune system of patients, which was based on blood lymphocyte count and albumin level (31). Albumin is a vital plasma protein of the body. It is synthesized exclusively by the liver with a median half-life of approximately 18–19 days (32). The function of albumin in maintaining normal activities of the human body is vital, especially in balancing intravascular fluid, binding and transporting various molecules and drugs, and antioxidant activities (32, 33). Albumin is also a crucial traditional laboratory marker for estimating malnutrition at the clinical level (33). Protein-energy malnutrition is closely related to negative prognosis implications among patients admitted to ICU (33). Hypoalbuminemia is a common issue in critically ill patients because inflammatory cytokines suppress the synthesis, and the concentration decreases via increased capillary permeability, hepatic failure, and renal or gastrointestinal tract losses (32, 34). Thus, in COVID-19, the condition is strongly related to widespread inflammation, and the reduction of albumin concentration has been well-reported (34, 35). In addition, the deficiency of the immune system, manifested as lymphopenia, is mentioned as one of the features of the COVID-19 infection (36). Many studies showed impaired cell-mediated immunity, decreased phagocytes, complement system, lymphocytes, and T cell count among patients with critical COVID-19 and low albumin levels (12, 37). The worse level of lymphopenia is related to the higher level of severity and mortality risk (38, 39). This issue has been explained in clinical studies emphasizing the role of cytokine storm contributed by the release of chemokines in COVID-19 (40).

### 4.3. Association of undernutrition and adverse treatment outcomes

Previous studies revealed several serious complications and adverse outcomes of critically ill COVID-19 patients, including hyper-inflammation, septic shock, acute respiratory distress syndrome (ARDS), and acute multi-organ injury (41, 42). The association between undernutrition assessed by GLIM, PNI, and detrimental prognostic of severe COVID-19 patients was presented in our analysis. This finding is consistent with previous studies that revealed a higher risk of in-hospital mortality and adverse treatment outcomes among malnutrition patients with COVID-19 and other severe diseases (6, 29). One of the main factors causing nutritional disturbances in severe COVID-19 patients is inadequate intake due to symptoms such as anorexia, nausea, vomiting, regurgitation and other gastrointestinal issues (delayed gastric emptying, diarrhea, constipation) that may facilitate the deterioration of the disease condition (43). On the other hand, the increased metabolic rate and enhanced gluconeogenesis, proteolysis, and fat oxidation can contribute to endocrine disorders and acute inflammation responses (44). Applying treatment interventions such as mechanical ventilation, antiviral, and broad-spectrum antibiotics medicines may give rise to the deterioration of digestive system function (27). Therefore, the progress of the disease can be accelerated by severe malnutrition and other pathophysiological mechanisms.

This study also found that patients with above-target FBG levels were more likely to have higher APACHE scores associated with adverse treatment results. In addition, FBG had good diagnostic ability in predicting patients with severe outcomes. Previous research presented similar findings, in which hyperglycemia was a common condition among critically ill patients with and without COVID-19 and was strongly correlated with hospitalized mortality (45, 46). There are several mechanisms to explain the hyperglycemia condition in severe COVID-19 patients. Firstly, hyperglycemia results from an intense cytokine storm, which covers a range of inflammatory and immune reactions (45, 46). Secondly, complex causes are impaired insulin secretion, worsening glucose disposal, pre-existing undiagnosed diabetes, or changes in metabolic and inflammatory homeostasis modification (45, 47). Thus, elevated blood glucose may increase the ability of respiratory failure, comorbidity infection, and death.

Several implications for ameliorating the severe conditions and mortality rate among COVID-19 patients can be put forward from the findings of this study. Since nutritional risk was a significant index to predict outcomes of severe COVID-19 cases, screening for malnutrition should be carefully conducted as a required examination at ICU admission and regularly during hospital stays. If an individual is defined as having malnutrition and at risk of other nutrition-related complications, appropriate nutritional care should be implemented, such as oral nutritional supplements or enteral nutrition and parenteral nutrition, depending on patients’ tolerance and condition (8). However, patients admitted to the ICU might not have sufficient information on medical history regarding weight loss or anthropometric measurements and muscle mass assessment. Thus, a simple and quick screening tool with few additional steps and high accuracy, such as PNI, should be considered to promptly define the malnutrition of severe COVID-19 patients before the entire assessment process is set up.

### 4.4. Strengths and limitations

Our research is one of the first studies evaluating the nutritional risk using multiple scales among COVID-19 patients admitted to the ICU to enhance the treatment outcomes. Nevertheless, some limitations should be acknowledged. We excluded patients with insufficient nutritional assessments in medical records, which may lead to selection bias for severe patients. In addition, information on weight loss and reduction of nutrient intake within the reference time was self-reported by the main caregivers, and possible recall bias may occur. Additionally, parameters of cytokine storm, including IL-6, ferritin, and D-dimer, were not considered. Convenience sampling can lead to selection bias because of the skewed or unrepresentative sample population. Finally, we applied the retrospective cross-sectional design, which hindered us from establishing the causal relationship between nutritional risk and the prognosis of COVID-19 patients.

## 5. Conclusion

In conclusion, a relatively high percentage of severe patients infected with COVID-19 experienced malnutrition. Individuals at higher risk of malnutrition and high FBG were more likely to have more detrimental treatment outcomes. In addition, non-invasive nutritional risk assessment scales, such as PNI and NRS, were good prognostic parameters regarding adverse outcomes of COVID-19. Regular nutritional screening and personalized appropriate nutritional care strategies are necessary to meet the patient’s nutrient needs and avoid other nutrition-related complications.

## Data availability statement

The raw data supporting the conclusions of this article will be made available by the authors, without undue reservation.

## Ethics statement

The studies involving humans were approved by the Committee of Saint Paul Hospital, code number 645/QĐ-BVĐKXP. The studies were conducted in accordance with the local legislation and institutional requirements. The participants provided their written informed consent to participate in this study.

## Author contributions

LHTN, AKD, TVT and HTL contributed to conception and design of the research. DATD, LBTN, AMT, TDD, TBN, TTN, and BHN contributed to data acquisition. LHTN, AKD, TVT, HTP, DATD, LBTN, AMT, TDD, TBN, and BHN contributed to the analysis and interpretation of the data. LHTN, AKD, TVT, HTP, HTL drafted the manuscript. LHTN, AKD, BHN, and HTL critically revised the manuscript. All authors agreed to be fully accountable for ensuring the integrity and accuracy of the work and read and approved the final manuscript.
